# A Distinct Intestinal Domination Fingerprint in Patients Undergoing Allo-HSCT: Dynamics, Predictors and Implications on Clinical Outcomes

**DOI:** 10.3390/jcm14238351

**Published:** 2025-11-24

**Authors:** Alexandre Soares Ferreira Junior, Danielle Amanda Niz Alvarez, Larissa da Silva Souza, Nathalia Linares Silva, Luiza Dias Machado, Welinton Yoshio Hirai, Rozana Mesquita Ciconelli, Joao Victor Piccolo Feliciano, Iago Colturato, George Maurício Navarro Barros, Phillip Scheinberg, Nelson Jen An. Chao, Gislane Lelis Vilela de Oliveira

**Affiliations:** 1 Laboratory of Immunomodulation and Microbiota, Department of Genetics, Microbiology and Immunology, Institute of Biosciences, Sao Paulo State University, Botucatu 18618-970, SP, Brazil; alexandre.soares@unesp.br (A.S.F.J.); danielle.alvarez@unesp.br (D.A.N.A.); larissa.silva-souza@unesp.br (L.d.S.S.); nathalia.linares@unesp.br (N.L.S.); luiza.d.machado@unesp.br (L.D.M.); 2 Department of Epidemiology and Biostatistics, Hospital de Amor de Barretos, Barretos 14784-400, SP, Brazil; welinton.hirai@hospitaldeamor.com.br; 3 Departamento de Pesquisa da BP—A Beneficência Portuguesa de São Paulo, São Paulo 01323-001, SP, Brazil; rozana.ciconelli@bp.org.br (R.M.C.); scheinbp@gmail.com (P.S.); 4 Fundação Faculdade Regional de Medicina de São José do Rio Preto (FUNFARME), São José do Rio Preto 15090-000, SP, Brazil; joao.feliciano@hospitaldebase.com.br; 5 Hospital Amaral Carvalho, Jaú 17210-070, SP, Brazil; iago_colt@hotmail.com; 6 Fundação Pio XII—Hospital de Câncer de Barretos, Barretos 14784-400, SP, Brazil; georgenavarrobr@yahoo.com.br; 7 Division of Hematologic Malignancies and Cellular Therapy, Duke University School of Medicine, Durham, NC 27710, USA; nelson.chao@duke.edu

**Keywords:** hematopoietic stem cell transplantation, Graft-versus-Host-Disease, intestinal microbiome, intestinal domination, clinical outcomes

## Abstract

**Background**: Although *Enterococcus* domination has been extensively evaluated in the context of allogeneic hematopoietic stem cell transplantation (allo-HSCT), the prevalence and clinical implications of other dominant genera remain poorly understood. **Objective:** In this study, we sought to determine the dynamics, predictors and clinical implications of intestinal domination in Brazilian patients undergoing allo-HSCT. **Methods:** In a prospective study of four Brazilian centers, fecal specimens were collected longitudinally prior to allo-HSCT until six months post-transplantation. To identify intestinal domination, we performed 16S rRNA gene sequencing using the Illumina platform. We then evaluated the impact of intestinal domination on overall survival and acute Graft-versus-Host-Disease (aGvHD) incidence. Finally, to identify predictors of intestinal domination, we performed a logistic regression model. **Results:** A total of 192 fecal specimens were collected from 69 patients. No significant changes in alpha or beta diversity were observed over the course of allo-HSCT. Among the 192 specimens, 131 (68%) presented intestinal domination. The top four dominant genera were *Bacteroides*, *Akkermansia*, *Phascolarctobacterium*, and *Escherichia-Shigella*. No significant associations were found between domination by these genera and either overall survival or aGvHD incidence. Furthermore, no patient-level characteristics, including age, sex, underlying disease, conditioning regimen, or stem cell source, reliably predicted intestinal domination. **Conclusions:** Our findings reveal a unique intestinal domination fingerprint in Brazilian patients and highlight the importance of geographic context in interpreting microbiota–outcome associations in allo-HSCT settings.

## 1. Introduction

Allogeneic hematopoietic stem cell transplantation (allo-HSCT) is a curative therapy for several malignant and non-malignant diseases [1,2,3,4,5,6]. Despite allo-HSCT’s potential benefits, life-threatening complications such as neutropenic fever, relapse and acute Graft-versus-Host-Disease (aGvHD) remain major barriers to successful outcomes [1,2,3,4,5,6]. The clinical success of allo-HSCT relies on the identification and mitigation of key variables with prognostic significance. Among these prognostic variables that can shape the course of the allo-HSCT, a key player seems to be the intestinal microbiota [7,8,9]. The intestinal microbiota plays a multifaceted role in human physiology, contributing to the preservation of the gut barrier integrity, producing key metabolites to maintain gut homeostasis, and shaping immune responses through complex crosstalk [10]. Through these mechanisms, the intestinal microbiota can shape the development of clinical outcomes over the allo-HSCT. For instance, aGvHD, which is the leading cause of non-relapse mortality, has been linked to specific patterns of intestinal microbiota disruption (“intestinal dysbiosis”) [11,12,13,14,15]. A decreased intestinal diversity (an index that measures intestinal microbiota variety [richness] and balance [evenness]) over the engraftment period has been associated with an approximately eight-fold increase in the risk of aGvHD [12]. Another intestinal dysbiosis feature frequently reported is the decreased abundance of SCFA (short-chain-fatty-acid)-producing bacteria, which has been associated with infections, transplant-related mortality, and overall survival [16,17,18]. These and other studies outline the impact of specific intestinal dysbiosis features in the context of allo-HSCT.

In patients undergoing allo-HSCT, another key feature of intestinal dysbiosis is the expansion of a single microbiota genus resulting in intestinal domination. Intestinal domination is a frequent dysbiosis feature in allo-HSCT, occurring in 28% to 80% of patients [19,20,21,22,23,24]. Although intestinal domination is common, the specific genus driving this event may vary across studies. For example, while *Enterococcus* was the most common dominant genus in the microbiota of patients from four different centers, *Streptococcus* and *Bacteroides* were predominant in other allo-HSCT studies [19,21,24]. Thus, although intestinal domination is consistently present in allo-HSCT studies, distinct domination fingerprints may emerge across different cohorts.

Across different cohorts, understanding how these distinct domination fingerprints may have prognostic significance is a key step to optimizing stratification and intervention strategies. Nevertheless, the available evidence has largely been focused on *Enterococcus* domination [20,21,22,23,24]. For instance, *Enterococcus* domination has been linked to significantly reduced overall survival, GvHD severity, and increased risk of bloodstream infections (BSI) [21,23,24]. However, how other dominant genera may impact clinical outcomes remains poorly understood.

Therefore, in this study, we sought to provide a comprehensive overview of the dynamics, predictors, and clinical implications of intestinal domination events in a cohort of Brazilian patients undergoing allo-HSCT. As shown in the subsequent sections of this manuscript, we identified a unique domination fingerprint in our cohort and conclude that the prognostic significance of intestinal domination during allo-HSCT may vary according to the dominant genera and the population being evaluated.

## 2. Patients, Material and Methods

### 2.1. Study Design, Ethical Aspects and Sample Collections

This is an ongoing, observational, multicenter, prospective, cohort study of patients undergoing allo-HSCT, which was approved by the Research Ethics Committee of Sao Paulo State University (process number 5.138.190/2021) and conducted according to the guidelines of the Declaration of Helsinki. The inclusion criteria were patients ≥ 12 years old undergoing allo-HSCT who provided at least one stool sample during the procedure and had clinical data available in REDCap. The exclusion criteria were: (1) cord blood transplant recipients, (2) patients who withdraw from the study, and (3) patients who did not provide stool samples over the 6-month follow-up. Antibiotic administration, nutritional status or presence of comorbidity was not considered as exclusion criteria. All participants signed an informed consent form at the beginning of the study. For patients <18 years old, both the patient and their legal representative agreed to participate in the study and signed consent forms.

Stool samples were collected at four different transplant centers (Hospital de Base of the Fundação Faculdade Regional de Medicina [HB-FUNFARME], Hospital Amaral Carvalho [HAC], Hospital de Cancer de Barretos [HCB] and Hospital Beneficência Portuguesa de São Paulo [BP]) and stored at −80 °C. Stool samples were collected longitudinally at 7 time points: prior to conditioning regimen (D−7), at the day of stem cell infusion (D0), 30 days after stem cell infusion (D+30), D+60, D+90, D+180, and at acute GvHD diagnosis. Although all efforts were made to collect the specimens at these exact time points, some variability occurred due to the timing and frequency of bowel movement patterns. Additionally, some samples were not collected from patients who were discharged from the original institution to continue follow-up in their hometown or, in some cases, when the patient was critically ill.

### 2.2. Intestinal Microbiota 16S Sequencing and Bioinformatics Pipeline

DNA was extracted from 200 mg of fecal samples by using QIAamp Fast DNA Stool Mini Kit (Qiagen, CA, USA), according to the manufacturer’s protocol. DNA was then quantified by Qubit dsDNA HS Assay Kit (Thermofisher, Waltham, MA, USA). The 16S rRNA V3-V4 regions were then amplified in a two-step PCR protocol using the following pre-determined primers—341F: 5′-TCGTCGGCAGCGTCAGATGTGTATAAGAGACAGCCTACGGGNGGCWGCAG, and 805R 5′- GTCTCGTGGGCTCGGAGATGTGTATAAGAGACAGGACTACHVGGGTATCTAATCC. PCR reactions used 2X Platinum SuperFi II PCR Master Mix (Thermofisher) and involved a denaturation at 98 °C for 30 s followed by 30 cycles of: 98 °C for 10 s, 55 °C for 10 s, and 72 °C for 30 s. Sequencing was done in the Illumina NextSeq 1000 platform (San Diego, CA, USA). A total of 45,590,208 reads were generated (average ~228,000 per sample) and underwent adapter removal using the Cutadapt v2.6 [25]. Then, the reads underwent quality filtering, denoising, chimera removal and amplicon sequence variant (ASV) inference using DADA2 v.1.26.0 [26]. A total of 17,934,483 reads (average ~89,700 per sample) passed all filtering and were used in downstream analyses. Taxonomic classification was performed using the Classify-sklearn naive Bayes classifier as implemented in Qiime2 (q2-feature-classifier plugin), which was trained on the SILVA SSU v138.1 NR 99 database [27,28]. The relative abundance of different ASVs in each sample was analyzed in Qiime2 and plotted with the taxa plugin (barplot function). Alpha and beta diversity indexes as well as statistical tests for significant differences across experimental groups were computed using the Qiime2 diversity plugin. The normalization method used in the study was rarefaction to 50,000 reads per sample (Qiime2 diversity plugin, alpha-rarefaction function; see Supplementary Figure S1). Tables were developed to compute the abundance of reads assigned to each ASV, which were then aggregated to the genus level for analyses. The metadata from this study have been submitted to the NCBI Sequence Read Archive (BioProject PRJNA1357096).

### 2.3. Statistical Analysis

Intestinal domination was defined as the relative abundance ≥ 30% of any specific genera within each stool sample [19,21,23,24,29]. This threshold was selected to align with prior allo-HSCT studies and ensure consistency with global definitions of intestinal domination. This analysis was focused on genera because this is generally the most specific level at which 16S sequencing can provide reliable classification [17]. We first generated descriptive statistics separated by intestinal domination status. Tests of associations between intestinal domination status and variables were examined by chi-square or Fisher’s exact test for categorical variables and Wilcoxon rank-sum test for continuous variables. Consistent with prior studies evaluating intestinal domination, the outcomes of interest were overall survival and cumulative incidence of aGvHD and severe aGvHD (grade 2–4) [17,21,24,30]. Overall survival was analyzed using the Kaplan–Meier methodology and survival curves were compared using the log-rank test. The time to event was calculated from D0 (day of stem cell infusion) to the date of death by any cause, aGvHD or last follow-up. Acute GvHD was diagnosed clinically, confirmed pathologically by biopsy, whenever possible, and graded per the MAGIC criteria [30]. Similar to prior studies, the onset of aGvHD was determined based on the onset of symptoms (clinical suspicion) [15]. A Cox regression analysis was used to evaluate the association between intestinal domination and aGvHD. A multivariate Cox regression analysis was performed to evaluate the association between intestinal domination and overall survival, as well as the cumulative incidence of GvHD, adjusting for potential confounders (age, sex, center, underlying diagnosis, conditioning regimen, donor sex, stem cell source, and donor type). This multivariate analysis was not conducted for severe GvHD due to the relatively low number of patients. Univariate logistic regression models were used to identify predictors of intestinal domination (see Supplementary Methods for details). A sensitivity analysis was performed specifically for overall survival to identify the optimal cutoff value for defining intestinal domination (expressed as relative abundance, %). For each potential threshold, samples were classified into “dominant” and “non-dominant” groups, which were then compared using Cox proportional hazards regression. A post hoc power analysis using the survivalpwr package in R was performed to evaluate whether the small sample size may have contributed to the significant association observed in our analysis, potentially indicating instability of the effect estimate [31]. All analyses were performed using the software R. A *p*-value of <0.05 was considered statistically significant.

## 3. Results

### 3.1. Diversity Metrics and Prevalence of Intestinal Domination

**Figure 1:**
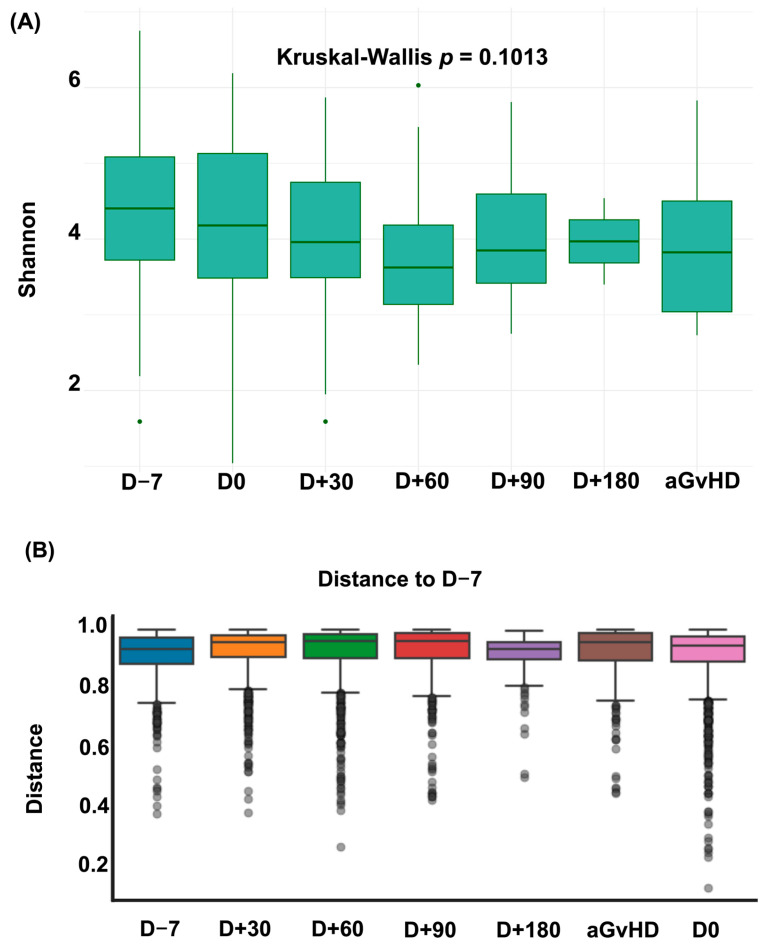
Dynamics of Alpha and Beta Diversity over the allo-HSCT. (**A**) Shannon Index over the allo-HSCT. (**B**) Bray–Curtis dissimilarity relative to pre-conditioning sample (D − 7). Each box represents the distribution of dissimilarities across patients at each time point. Higher distances indicate greater shifts in microbiota composition relative to baseline. aGvHD = acute graft versus host disease; D = Day.

During the study period, 69 patients undergoing allo-HSCT provided 192 stool samples. The proportion of samples at each time point is shown in Supplementary Table S1. No significant changes in alpha or beta diversity were observed over the course of allo-HSCT (see Figure 1).

**Figure 2:**
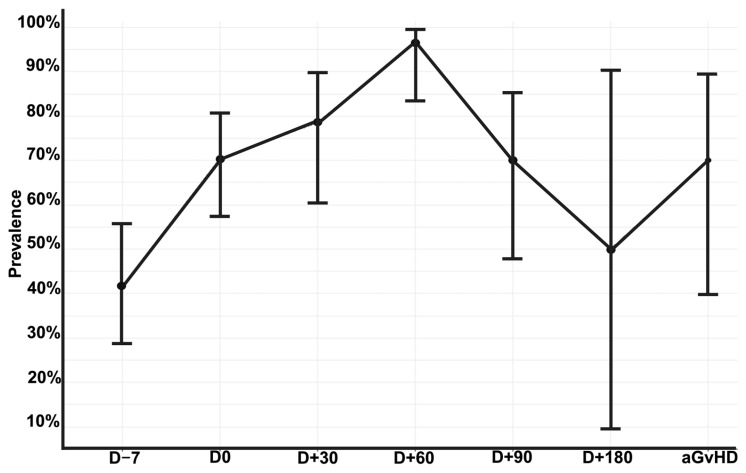
Prevalence of intestinal domination by any genus over the allo-HSCT. Error bars representing 95% confidence interval calculated using the Wilson score method for proportions to account for small sample sizes at late time points. Allo-HSCT = Allogeneic hematopoietic stem cell transplantation. aGvHD = acute Graft-versus-Host Disease; D = Day. Edited with BioRender.com, Soares Ferreira Junior A. (2025) https://BioRender.com/zd0vcep (accessed on 3 November 2025).

At the patient level, the prevalence of intestinal domination at any time point was 78.2% (*n* = 54/69). Among the 192 stool samples, 68.2% (*n* = 131/192) exhibited an intestinal domination event at any time point. The prevalence of intestinal domination at each time point is shown in Figure 2. The lowest prevalence occurred in samples collected prior to the conditioning regimen (41.6%; *n* = 20/48). The highest prevalence occurred in samples collected 60 days after allo-HSCT (96.6%; *n* = 29/30). At the time of aGvHD diagnosis, 70% of patients (*n* = 7/10) presented intestinal domination by a single genus. Additionally, among the 131 samples with intestinal domination, 12.9% (*n* = 17/131) had concurrent domination by two distinct genera (see Supplementary Table S2).

**Figure 3:**
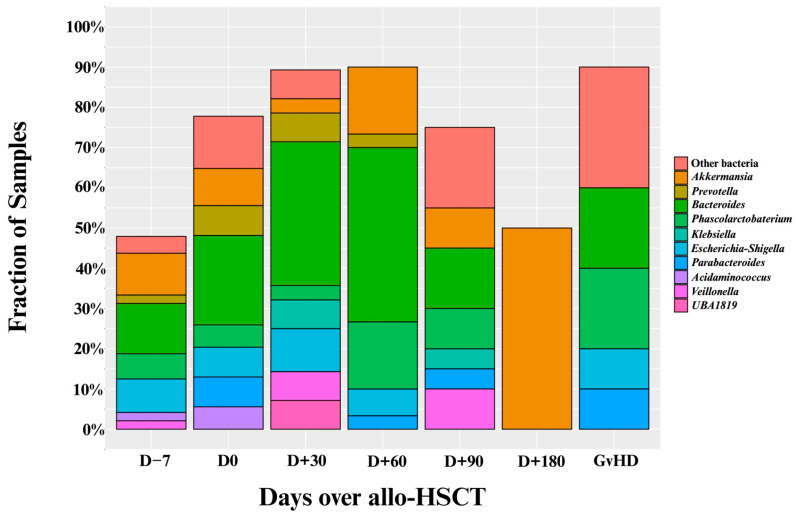
Proportion of intestinal domination events by specific genera over the allo-HSCT. Allo-HSCT = Allogeneic hematopoietic stem cell transplantation. Edited with BioRender.com, Soares Ferreira Junior A. (2025) https://BioRender.com/xzrbtuf (accessed on 3 November 2025).

The specific genera responsible for these intestinal domination events are shown in Figure 3. Overall, most of these events resulted from the expansion of four genera: (1) *Bacteroides* (*n* = 46), (2) *Akkermansia* (*n* = 19), (3) *Phascolarctobacterium* (*n* = 16), and (4) *Escherichia–Shigella* (*n* = 14; see Figure 2). Interestingly, only one sample (0.5%) showed *Enterococcus* domination (see Supplementary Table S3).

### 3.2. Patient Characteristics by Intestinal Domination Status

Patient demographic information classified by intestinal domination status is shown in Table 1. No statistically significant differences were found for any of the demographic characteristics (age, weight, height, and sex). Additionally, no statistically significant differences were found for any allo-HSCT-related variable (prior allo-HSCT, stem cell source, donor type, and conditioning regimen).

### 3.3. Analysis of Clinical Outcomes

**Figure 4:**
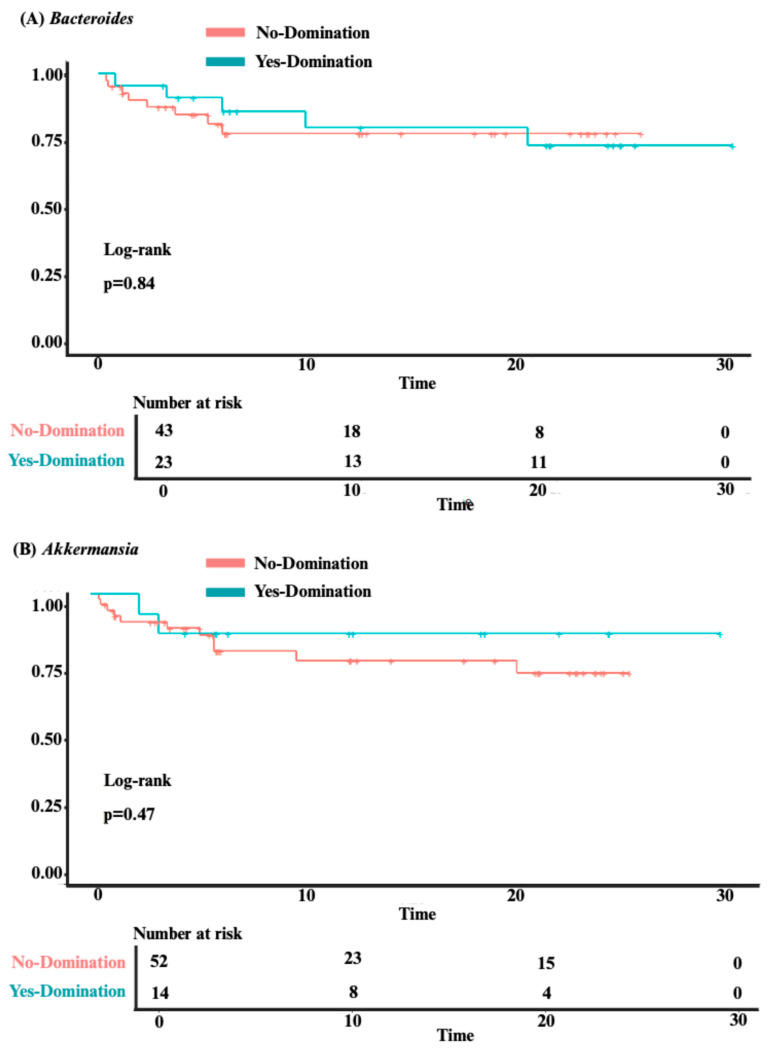

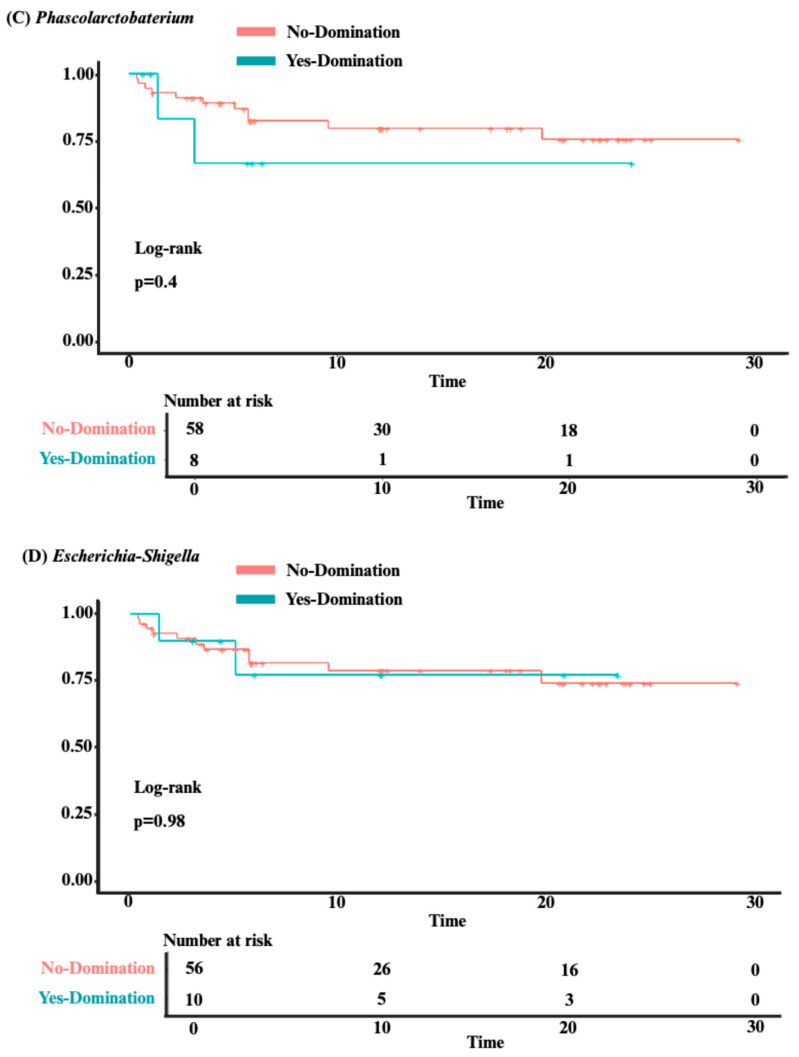
The impact of intestinal domination on overall survival. (**A**) *Bacteroides*. (**B**) *Akkermansia*. (**C**) *Phascolarctobacterium*. (**D**) *Escherichia-Shigella*.

**Figure 5:**
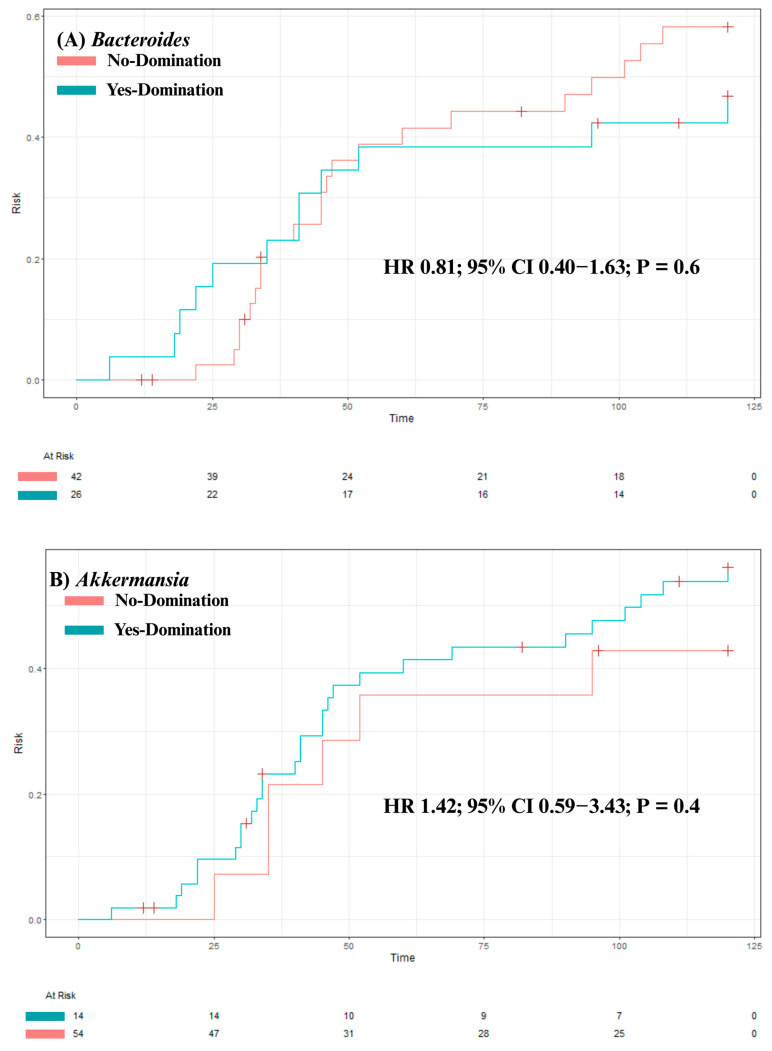

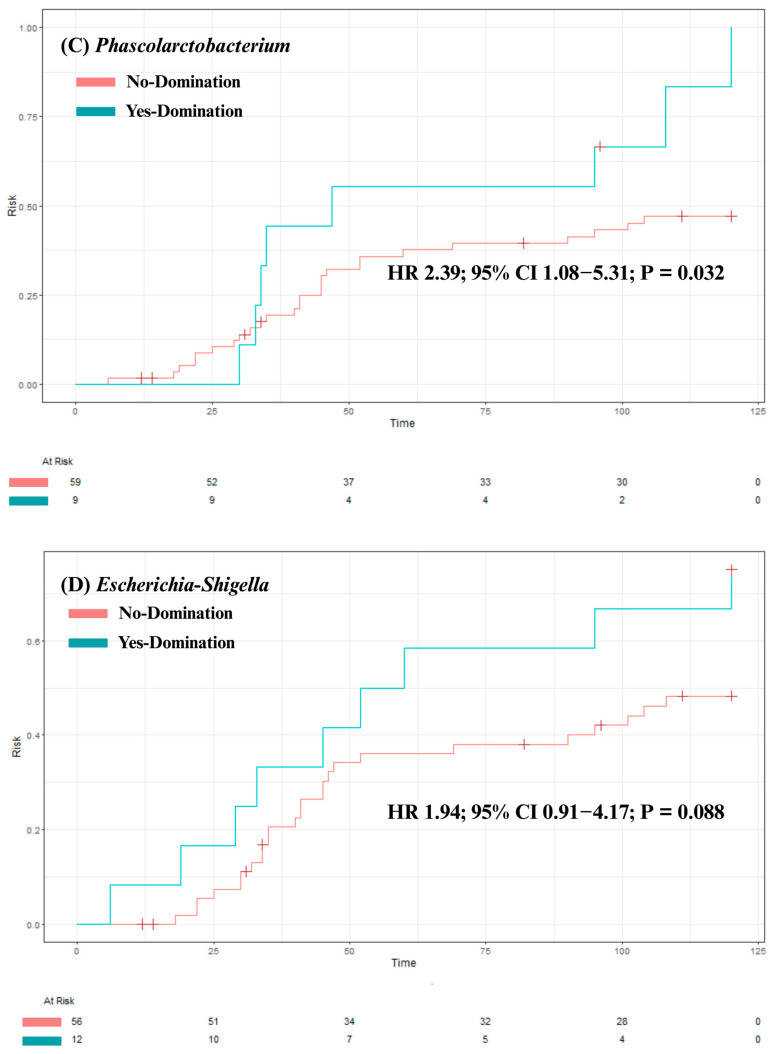
The impact of intestinal domination on the cumulative incidence of aGvHD. (**A**) *Bacteroides*. (**B**) *Akkermansia*. (**C**) *Phascolarctobacterium*. (**D**) *Escherichia-Shigella*. HR = Hazard Ratio. CI = Confidence interval.

Our analysis identified no significant association between overall survival and intestinal domination by the four most frequently identified genera—*Bacteroides* (*p* = 0.84), *Akkermansia* (*p* = 0.47), *Phascolarctobacterium* (*p* = 0.4), and *Escherichia-Shigella* (*p* = 0.98; see Figure 4A–D). Although univariate analysis revealed a significant association between *Phascolarctobacterium* domination and cumulative incidence of aGvHD (HR 2.39 [1.08–5.31]; *p* = 0.032), this was no longer significant after adjusting for confounders (age, sex, center, underlying diagnosis, conditioning regimen, donor sex, stem cell source, and donor type) in a multivariate analysis (HR 1.75 [0.73–4.20]; *p* = 0.20). Also, post hoc power analysis showed 67% power to detect the observed effect size (HR = 2.39) for aGvHD, indicating that the significant association identified in our study may be unstable due to the small sample size and should be interpreted with caution. For the other three genera, no significant associations were found between domination and cumulative incidence of aGvHD (*Bacteroides:* HR = 0.81 [0.40–1.63]; *p* = 0.6; *Akkermansia*: HR = 1.42 [0.59–3.43]; *p* = 0.4; and *Escherichia-Shigella:* HR = 1.94 [0.91–4.17]; *p* = 0.088; see Figure 5A–D). Finally, no significant association was observed between domination by any of the four genera and the cumulative incidence of severe aGvHD—*Bacteroides*: HR = 1.18 [0.44–3.15]; *p* = 0.7; *Akkermansia*: HR = 0.54 [0.16–1.89]; *p* = 0.3; *Phascolarctobacterium:* HR = 0.64 [0.18–2.26]; *p* = 0.5; and *Escherichia-Shigella*: HR = 1.66 [0.54–5.14]; *p* = 0.4 (see Supplementary Figure S2A–D). Multivariate analyses assessing the associations between intestinal domination and overall survival, as well as the cumulative incidence of GvHD, are presented in Supplementary Table S4. The sensitivity analysis performed to identify the optimal cutoff value for defining intestinal domination corroborated the 30% threshold for *Phascolarctobacterium*, and are shown in Supplementary Figure S3A–D.

### 3.4. Predictors of Intestinal Domination

The univariate logistic regression model of predictors of intestinal domination by any genera and by *Bacteroides*, *Akkermansia*, *Phascolarctobacterium,* and *Escherichia-Shigella* is shown in Table 2. None of the variables were associated with intestinal domination. Given the absence of statistically significant variables in the univariable model, a multivariable model was not performed.

## 4. Discussion

In this multicenter, prospective, observational study, we outlined the dynamics, predictors, and clinical implications of intestinal domination in Brazilian patients undergoing allo-HSCT. We first found that intestinal domination is a prevalent dysbiosis fingerprint occurring in 78.2% of patients. Then, we identified a unique pattern of domination in Brazilian patients characterized by an extremely low prevalence of *Enterococcus* domination (only 1.4% of patients). In this fingerprint, most intestinal domination events were driven by the expansion of *Bacteroides*, *Akkermansia*, *Phascolarctobacterium*, and *Escherichia-Shigella.* Although this domination fingerprint was frequent in our cohort, it did not appear to significantly impact key clinical outcomes such as overall survival and aGvHD incidence. Finally, none of the patient or allo-HSCT-related characteristics included in our analysis predicted intestinal domination.

In our analysis, the most interesting finding is that although intestinal domination occurred in 78.2% of patients, this was rarely driven by *Enterococcus* expansion (only one patient). This extremely low prevalence of *Enterococcus* domination (1.4%) differs from the current literature. In prior studies examining intestinal domination in patients undergoing allo-HSCT, *Enterococcus* has been identified as a key driver of domination events, occurring in 36% to 65% of patients [9,19,21,22,23,24]. On the higher end of the spectrum of reported *Enterococcus* domination is a 65% domination prevalence reported in a study including 1325 patients with 9049 stool specimens [21]. In this study, the genus *Enterococcus* was the most commonly observed to dominate the microbiota in patients from the four different participating centers [21]. On the lower end of the spectrum is a 36% *Enterococcus* domination prevalence occurring in a study including 98 patients with 681 stool specimens [24]. In this study, the median time between transplant date and first stool sample with *Enterococcus* domination was 22 days (IQR 6.75–84.25) [24]. It is important to note, however, that each of these studies used distinct sampling time points, which may hinder an accurate comparison across studies [9,19,21,22,23,24]. Also, antibiotic use, a known disruptor of intestinal domination, was not collected across all centers and, therefore, excluded from this analysis. This represents a key limitation, as center-specific antibiotic practices (e.g., levofloxacin prophylaxis; see Supplementary Table S5) may explain the low *Enterococcus* prevalence identified in our study. Nevertheless, compared to these studies, our findings suggest that *Enterococcus* may not be a key genus driving intestinal domination in Brazilian patients. Therefore, future studies should investigate which other genera are driving intestinal domination in this cohort.

Our findings identified that important genera driving intestinal domination in Brazilian patients are *Bacteroides*, *Akkermansia*, *Phascolarctobacterium*, and *Escherichia-Shigella*. In our cohort, these four genera were responsible for most of the domination events. While *Bacteroides* and *Akkermansia* have been reported as dominant genera in prior studies, the high frequency of *Phascolarctobacterium* and *Escherichia-Shigella* domination is not well demonstrated in the prior literature [9,19,20,21,22,23,24]. The dynamics of intestinal domination was evaluated in 100 patients with 603 stool specimens [19]. This study demonstrated an interesting compositional shift [19]. While *Bacteroides* was the most common dominant genus before conditioning, at the time of engraftment, intestinal domination occurred predominantly due to pathogenic genera such as *Enterococcus*, *Klebsiella* and *Escherichia-Shigella* [19]. In another study including 98 patients undergoing allo-HSCT, 681 stool specimens were analyzed [24]. The most frequent genera responsible for intestinal domination, in decreasing order, were as follows: (1) *Streptococcus* (42%), (2) *Enterococcus* (36%), and (3) *Bacteroides* (38%) [24]. Other important dominant genera reported in this study were *Akkermansia*, *Blautia* and *Lactobacillus*, each of which was associated with intestinal domination in approximately 28% of patients. A similar profile was also identified in another study including 94 patients with 439 stool specimens [23]. In this study, *Enterococcus* was the most frequent dominating genus (40%), followed by *Streptococcus* (37%) [23]. When compared to these previous studies, our findings suggest that Brazilian patients undergoing allo-HSCT may exhibit a unique intestinal domination fingerprint.

This unique intestinal domination fingerprint likely reflects a combination of factors that modulate the intestinal microbiota during allo-HSCT. First, it may be partly explained by distinct dietary habits in Brazilian patients (e.g., high intake of fiber, cereals, rice and yogurt) [19,32,33]. High fiber consumption, for instance, has been associated with greater intestinal microbial diversity and increased production of SCFAs [33]. Conversely, unhealthy dietary patterns characterized by high sugar or fat intake have been linked to shifts in microbial composition and decreased intestinal diversity [34]. Second, this fingerprint may result from center-specific antibiotic practices and distinct *Enterococcus* resistance and colonization profiles observed across countries [22,23,35]. Finally, the persistence of *Bacteroides* domination (a common feature in healthy individuals) during allo-HSCT, may also suggest a more resilient microbiota in our cohort [24,33,36,37]. Microbiota resilience refers to the ability of the microbial community to rapidly return to its original state despite major perturbations that may occur during allo-HSCT (e.g., antibiotic exposure, conditioning regimens, infections, or dietary changes) [33,34]. This hypothesis is further supported by our longitudinal analysis of intestinal diversity over the allo-HSCT, which showed no significant differences between baseline and post-allo-HSCT samples (see Figure 1). Therefore, multiple factors may contribute to this distinctive intestinal domination fingerprint observed in Brazilian patients, and future studies incorporating detailed dietary, antibiotic, and functional experiments assessing microbiota resilience are warranted.

Whether this intestinal domination fingerprint identified in our analysis has prognostic significance was the next question we tried to answer in this study. In our attempt to evaluate the prognostic significance, we evaluated overall survival and aGvHD, which are key clinical outcomes that have been linked with *Enterococcus* domination [20,21,23,24]. For example, in the aforementioned study including 1,325 patients undergoing allo-HSCT, *Enterococcus* domination was associated with an approximately two-fold decrease in overall survival (HR 1.97; 95% CI 1.45–2.66; *p* < 0.001) [21]. This finding remained significant in a multivariate analysis adjusted for graft source, age, conditioning intensity, gender, and underlying disease (HR 2.06; 95% CI 1.50–2.82; *p* < 0.0001) [21]. In this same study, *Enterococcus* domination was also significantly associated with GvHD severity in both univariate (HR 1.44; 95% CI 1.10–1.88; *p* < 0.01) and multivariate (HR 1.32; 95% CI 1.00–1.75; *p* < 0.05) analyses [21]. It is worth noting that few studies in the literature have specifically analyzed the clinical implications of intestinal domination by some of the genera identified in our cohort. *Bacteroides* and *Akkermansia* domination, for example, was not significantly associated with overall survival (*p* = 0.08 and *p* = 0.14) in a prior study with 98 patients [24]. This is further corroborated by our findings, demonstrating that intestinal domination by *Bacteroides*, *Akkermansia*, *Phascolarctobacterium* and *Escherichia-Shigella* may not impact clinical outcomes. Thus, our findings suggest that the prognostic significance of intestinal domination may vary according to the dominant genera and the population being evaluated.

Although evidence directly addressing the prognostic impact of domination by these genera is lacking, prior studies have reported associations between their relative abundances and key clinical outcomes during allo-HSCT [4,24,38,39,40]. Some of these genera have been associated with aGvHD, overall survival, and relapse-free survival [38,39,40]. In a previous allo-HSCT study including 71 patients, the relative abundance of *Bacteroides* was significantly lower in patients with aGvHD compared to those without (*p* < 0.05) [38]. In that same study, higher *Bacteroides* abundance was also associated with a reduced incidence of aGvHD (*p* = 0.0043), and improved aGvHD-free/relapse-free survival (*p* = 0.021) [38]. In another study including 37 patients with aGvHD, significantly higher abundances of *Escherichia–Shigella* and *Enterococcus* were observed compared to healthy controls. Additionally, patients with steroid-refractory aGvHD showed significantly reduced *Bacteroides* abundance compared to steroid-responsive patients (*p* = 0.007). Decreased *Akkermansia* abundance was also associated with aGvHD in another study including 150 patients (*p* < 0.05) [4]. Although less explored in the allo-HSCT context, prior studies have highlighted *Phascolarctobacterium* as a key genus associated with diseases such as diabetes through SCFA production [41,42,43]. In our analysis, although *Phascolarctobacterium* was significantly associated with aGvHD in the univariate analysis, this association did not remain significant in the multivariate model. Taken together, these studies suggest that the genera identified in our cohort may have prognostic relevance when evaluated as continuous measures of relative abundance. The lack of significant associations in our analysis may reflect (1) limited statistical power, and (2) the inherent loss of information caused by categorizing continuous microbial abundances into binary domination events. Therefore, future large prospective studies are needed to validate these findings and to strengthen the evidence surrounding the prognostic significance of this unique fingerprint.

Given the potential prognostic significance of intestinal domination, the final planned analysis was to identify predictors of intestinal domination in our cohort. Our findings demonstrated that no patient-level characteristics, including age, sex, underlying disease, conditioning regimen, or stem cell source, could reliably predict intestinal domination, although antibiotic use (a known microbiota disruptor) was not collected and therefore could not be evaluated. This finding is partially supported by previous studies [22,23]. With the aim to identify predictors of *Streptococcus* domination, a prior study with 94 patients evaluated the following variables: age, sex, underlying diagnosis, prior antibiotic use, conditioning regimen intensity, T-cell depleted graft, stem cell source, and fever [23]. In this analysis, none of the evaluated variables reliably predicted Streptococcus domination [23]. However, in this same study, *Enterococcus* domination was increased three-fold in patients receiving metronidazole (HR 3.38; 95% CI 1.65–6.73; *p* = 0.01) and in patients with acute leukemia (HR 3.22; 95% CI 1.60–6.94; *p* = 0.01) [23]. Similar findings were reported by another study with 46 patients undergoing allo-HSCT [22]. Among seven potential predictors (age, sex, donor source, graft type, conditioning regimen intensity, and TBI-containing regimen), only acute leukemia was associated with *Enterococcus* domination (HR 2.48; 95% CI 1.13–5.45; *p* = 0.024) [22]. When combined with our findings, these data suggest that patient-level variables may not be reliable predictors of intestinal domination. Additionally, they suggest that predictors of intestinal domination may vary depending on the specific genus involved. Given the prognostic significance associated with some of these dominant genera, future studies evaluating other potential predictors are warranted.

The primary strength of this study is its comprehensive approach to evaluating the dynamics, predictors, and clinical implications of intestinal domination in Brazilian patients undergoing allo-HSCT. Nevertheless, our study presents some limitations. First, although we had pre-specified time points for stool sample collection, many patients were unable to provide samples at later stages, which significantly reduced the number of samples at D + 90 and D + 180. These relatively small sample sizes limit our ability to draw definitive conclusions regarding late post-transplantation microbial dynamics and to determine the prognostic significance of this unique domination fingerprint. Additionally, we did not collect stool samples over the engraftment period (defined as the first day of absolute neutrophil count ≥ 500/μL for 3 consecutive days, typically occurring between D + 7 and D + 21), which has been analyzed by prior studies [9,19,20,21,22,23,24]. This period is associated with peak microbiota dysbiosis and *Enterococcus* expansion. Therefore, the lack of samples over this period hinders an accurate comparison with other studies. Furthermore, due to data collection limitations in some centers, we were unable to evaluate: (1) the impact of intestinal domination in bloodstream infections, and (2) the role of antibiotics as a predictor of intestinal domination. Additionally, given the relatively small number of samples, analyses exploring clinical correlates of concurrent domination or stratified analyses were not performed. Also, all participating centers in our study were located within a single Brazilian state (São Paulo), which may limit the generalizability of our findings. Finally, our study does not provide mechanistic interpretations. Notwithstanding these limitations, our study is an important milestone in defining the dynamics and implications of intestinal domination events in Brazilian patients undergoing allo-HSCT, identifying a unique domination fingerprint that establishes important preliminary observations for future mechanistic investigations.

## 5. Conclusions

In a cohort of Brazilian patients undergoing allo-HSCT, we identified a unique intestinal domination fingerprint characterized by an extremely low prevalence of *Enterococcus* expansion. Although intestinal domination was a prevalent dysbiosis feature occurring in 78.2% of patients, our findings showed that intestinal domination by *Bacteroides*, *Akkermansia*, *Phascolarctobacterium,* and *Escherichia-Shigella* was not significantly associated with overall survival and aGvHD incidence. This pattern contrasts with international cohorts, in which *Enterococcus* often predominates and correlates with adverse outcomes, suggesting that the prognostic significance of intestinal domination during allo-HSCT may differ according to population-specific factors such as antibiotic practices and diet. These findings highlight the importance of considering geographic and environmental context when interpreting microbiota–outcome associations and support the need for future multi-center studies to elucidate the factors driving these distinct domination fingerprints.

## Figures and Tables

**Table 1:** Baseline Demographic Characteristics by Intestinal Domination Status.

Variable	No-Intestinal Domination (*n* = 15)	Yes-Intestinal Domination (*n* = 54)	Total (*N* = 69)	*p*-Value
Age (years)				0.6
Mean (SD)	44 (19)	41 (15)	40 (16)	
Median (IQR)	40 (27–68)	42 (31–51)	40 (28–49)	
Range	18–74	12–71	12–73	
Weight (kg)				0.12
Mean (SD)	79 (16)	72 (17)	74 (17)	
Median (IQR)	79 (68–87)	68 (62–82)	74 (64–82)	
Range	48–110	43–130	43–130	
Height (cm)				0.091
Mean (SD)	170 (10)	166 (10)	167 (10)	
Median (IQR)	173 (160–178)	164 (158–174)	165 (160–175)	
Range	150–185	146–189	146–189	
Center				0.2
HB-FUNFARME	4 (27%)	5 (9.3%)	9 (13%)	
HAC	4 (27%)	18 (33%)	22 (32%)	
HCB	0 (0%)	8 (15%)	8 (12%)	
BP	7 (47%)	23 (43%)	30 (43%)	
Sex				0.2
Male	9 (60%)	22 (41%)	31 (45%)	
Female	6 (40%)	32 (59%)	38 (55%)	
Prior Allo-HSCT	0 (0%)	3 (6.4%)	3 (5.1%)	>0.9
Primary Disease				0.4
Acute Myeloid Leukemia	6 (40%)	23 (43%)	29 (42%)	
Acute Lymphoid Leukemia	1 (6.7%)	15 (28%)	16 (23%)	
Chronic Myeloid Leukemia	1 (6.7%)	2 (3.7%)	3 (4.3%)	
Hodgkin’s Lymphoma	1 (6.7%)	1 (1.9%)	2 (2.9%)	
Non-Hodgkin’s Lymphoma	0 (0%)	1 (1.9%)	1 (1.4%)	
Aplastic Anemia	2 (13%)	5 (9.3%)	7 (10%)	
Sickle Cell Disease	1 (6.7%)	2 (3.7%)	3 (4.3%)	
Other	3 (20%)	5 (9.3%)	8 (12%)	
Stem Cell Source				0.4
Peripheral Blood	9 (60%)	39 (72%)	48 (70%)	
Bone Marrow	6 (40%)	15 (28%)	21 (30%)	
Donor Type				0.11
Matched Related	3 (20%)	17 (31%)	20 (29%)	
Matched Unrelated	4 (27%)	3 (5.6%)	7 (10%)	
Mismatched Related	0 (0%)	3 (5.6%)	3 (4.3%)	
Haploidentical	7 (47%)	30 (56%)	37 (54%)	
Mismatched Unrelated	1 (6.7%)	1 (1.9%)	2 (2.9%)	
Donor Sex				0.4
Male	7 (47%)	33 (61%)	40 (58%)	
Female	8 (53%)	21 (39%)	29 (42%)	
Intensity of Conditioning Regimen				0.8
Ablative	4 (27%)	21 (39%)	25 (36%)	
Reduced Intensity	7 (47%)	20 (37%)	27 (39%)	
Nonmyeloablative	4 (27%)	12 (22%)	16 (23%)	
TBI-Conditioning Regimen	6 (40%)	29 (54%)	35 (51%)	0.3

Allo-HSCT = Allogeneic hematopoietic stem cell transplant; BP = Hospital Beneficencia Portuguesa de Sao Paulo; kg = kilograms; cm = centimeters; SD = Standard deviation; IQR = Interquartile range; TBI = Total body irradiation; HB-FUNFARME = Hospital de Base of Fundacao Faculdade Regional de Medicina; HAC = Hospital Amaral Carvalho; HCB = Hospital de Cancer de Barretos.

**Table 2:** Univariable Logistic Regression Model of Predictors of Intestinal Domination.

	Any Genus		*Bacteroides*		*Akkermansia*		*Phascolarctobacterium*		*Escherichia-Shigella*	
	OR (95%CI)	*p* Value	OR (95%CI)	*p* Value	OR (95%CI)	*p* Value	OR (95%CI)	*p* Value	OR (95%CI)	*p* Value
Age	0.99 (0.95–1.02)	0.46	0.99 (0.96–1.02)	0.67	0.99 (0.96–1.03)	0.73	1.03 (0.99–1.08)	0.18	1.00 (0.96–1.04)	0.88
BMI	0.98 (0.88–1.09)	0.69	1.01 (0.92–1.11)	0.81	0.99 (0.89–1.11)	0.90	1.05 (0.93–1.20)	0.41	0.94 (0.82–1.06)	0.32
Sex										
Female	-	-	-	-	-	-	-	-	-	-
Male	0.46 (0.14–1.45)	0.19	0.65 (0.24–1.75)	0.40	0.41 (0.10–1.41)	0.18	0.31 (0.04–1.39)	0.16	0.85 (0.23–2.99)	0.80
Conditioning regimen										
Reduced intensity	-	-	-	-	-	-	-	-	-	-
Myeloablative	1.75 (0.46–7.53)	0.42	1.20 (0.39–3.69)	0.75	2.33 (0.61–10.1)	0.23	1.14 (0.19–6.70)	0.88	1.14 (0.24–5.39)	0.86
Non-myeloablative	1.00 (0.25–4.47)	>0.99	1.08 (0.29–3.85)	0.91	1.38 (0.24–7.24)	0.70	1.92 (0.32–11.7)	0.46	2.00 (0.41–9.87)	0.38
TBI-Conditioning Regimen	1.74 (0.55–5.84)	0.35	1.57 (0.59–4.26)	0.37	1.38 (0.43–4.70)	0.59	1.25 (0.30–5.48)	0.76	0.97 (0.27–3.43)	0.96
Stem cell source										
Peripheral	-	-	-	-	-	-	-	-	-	-
Bone marrow	0.58 (0.18–1.98)	0.37	1.03 (0.35–2.93)	0.96	1.35 (0.37–4.58)	0.63	0.25 (0.01–1.50)	0.21	1.83 (0.48–6.60)	0.36
Underlying diagnosis										
Acute leukemia	-	-	-	-	-	-	-	-	-	-
Others	0.40 (0.12–1.33)	0.13	0.76 (0.25–2.20)	0.62	0.89 (0.22–3.10)	0.87	0.25 (0.01–1.50)	0.21	1.18 (0.28–4.29)	0.81

BMI = Body mass index; CI = Confidence interval; OR = Odds ratio; TBI = Total body irradiation.

## Data Availability

The metadata from this study have been submitted to the NCBI Sequence Read Archive (BioProject PRJNA1357096).

## Supplementary Materials

The following supporting information can be downloaded at: https://www.mdpi.com/article/10.3390/jcm14238351/s1, Table S1: Proportion of Samples in Each Timepoint; Table S2: Details of Samples with Concurrent Domination by Two Distinct Genera; Table S3. Number of Intestinal Domination Events per Genus; Table S4: Multivariate Analyses Assessing Associations Between Intestinal Domination and Clinical Outcomes; and Table S5. Antibiotic Practices in Each Institution; Figure S1: Rarefaction curves showing the number of observed features (ASVs) as a function of sequencing depth for all stool samples included in the study; Figure S2. The Impact of intestinal domination on the cumulative incidence of severe aGvHD; and Figure S3: Sensitivity analysis to identify the optimal cutoff value for defining intestinal domination in relation to overall survival. Supplementary Methods—Predictors of Intestinal Domination [22,23,44].
