# Supplementary material for: A Distinct Intestinal Domination Fingerprint in Patients Undergoing Allo-HSCT: Dynamics, Predictors and Implications on Clinical Outcomes
Source: J Clin Med. 2025 Nov 24;14(23):8351. doi: 10.3390/jcm14238351 (PMC12693016; doi:10.3390/jcm14238351)
Supplement: Supplementary file 1 [file jcm-14-08351-s001.zip › jcm-3866275-supplementary.pdf]

A Distinct Intestinal Domination Fingerprint in Patients Undergoing Allo-HSCT: Dynamics, Predictors and Implications on Clinical Outcomes

Alexandre Soares Ferreira Junior,[1] Danielle Amanda Niz Alvarez,[1] Larissa da Silva Souza,[1] Nathalia Linares Silva,[1] Luiza Dias Machado,[1] Welinton Yoshio Hirai,[2] Rozana Mesquita Ciconelli,[3] Joao Victor Piccolo Feliciano,[4] Iago Colturato,[5] George Maurício Navarro Barros,[6] Phillip Scheinberg,[3] Nelson J Chao,[7] Gislane Lelis Vilela de Oliveira[1*]

[1] Laboratory of Immunomodulation and Microbiota, Department of Genetics, Microbiology and Immunology, Institute of Biosciences, Sao Paulo State University, Botucatu, Brazil.
[2] Department of Epidemiology and Biostatistics, Hospital de Amor de Barretos, Barretos, Sao Paulo, Brazil.
[3] Departamento de Pesquisa da BP- A Beneficência Portuguesa de São Paulo, São Paulo, Brazil.
[4] Fundação Faculdade Regional de Medicina de São José do Rio Preto (FUNFARME), São José do Rio Preto, Sao Paulo, Brazil.
[5] Hospital Amaral Carvalho, Jaú, Sao Paulo, Brazil.
[6] Fundação Pio XII - Hospital de Câncer de Barretos, Barretos, Sao Paulo, Brazil.
[7] Division of Hematologic Malignancies and Cellular Therapy, Duke University School of Medicine, Durham, North Carolina, United States.
* Correspondence: gislane.lelis@unesp.br; 55 (14) 3880-0749; Botucatu, Sao Paulo, Brazil.

Supplementary Table 1. Proportion of Samples in each Timepoint

Timepoint	N (%)
**D-7**	48 (25.0%)
**D0**	54 (28.1%)
**D+30**	28 (14.6%)
**D+60**	30 (15.6%)
**D+90**	20 (10.4%)
**D+180**	2 (1.0%)
**aGvHD**	10 (5.2%)

aGvHD = acute graft *versus* host disease

Supplementary Table 2. Details of Samples with Concurrent Domination by Two Distinct Genera

Time Point	Number of Sample	Codomination Genera
D-7	3	*Escherichia-Shigella + Veillonella*
		Akkermansia + Bacteroides
		Akkermansia + Bacteroides
D0	4	*Akkermansia + Bifidobacterium*
		Clostridia_vadinBB60_group + UCG-004
		Acidaminococcus + Parabacteroides
		Bacteroides + Escherichia-Shigella
D+30	3	*Bacteroides + Fusobacterium*
		Klebsiella + Uncultured

		Prevotella + Veillonella
D+60	4	*Bacteroides + Uncultured*
		Bacteroides + Phascolarctobacterium
		Escherichia-Shigella + Phascolarctobacterium
		Bacteroides + Parabacteroides
D+90	1	*Veillonella + Uncultured*
D+180	0	N/A
GvHD	2	*Fusobacterium + Phascolarctobacterium*
		Bacteroides + Parabacteroides
D = Day; GvHD = Graft *versus* host disease; N/A = Not Applicable.		

Supplementary Table 3. Number of Intestinal Domination Events per Genus

Genus	Number of Samples
Bacteroides	46
Akkermansia	19
Phascolarctobacterium	16
Escherichia-Shigella	14
Prevotella	8
Parabacteroides	7
Veillonella	5
Acidaminococcus	4
Campylobacter	3
Fusobacterium	3
Klebsiella	3
Uncultured	3
Alistipes	2
Bilophila	2
Clostridia_vadinBB60_group	2
Dialister	2
UBA1819	1
Bifidobacterium	1
Enterococcus	1
Faecalibacterium	1
Haemophilus	1
Lactobacillus	1
Staphylococcus	1
UCG-004	1

Supplementary Table 4. Multivariate Analyses Assessing Associations Between Intestinal Domination and Clinical Outcomes

Genera	Overall Survival* HR [95% CI]; P value	aGvHD Cumulative Incidence* HR [95% CI]; P value

Bacteroides	1.56 [0.42-5.85]; P = 0.5	0.64 [0.29-1.40]; P = 0.3
Akkermansia	1.27 [0.16-10.2]; P = 0.8	0.41 [0.14-1.26]; P = 0.12
Phascolarctobacterium	1.66 [0.15-18.3]; P = 0.7	1.66 [0.58-4.76]; P = 0.3
Escherichia-Shigella	0.52 [0.07-3.91]; P = 0.5	1.68 [0.68-4.14]; P = 0.3
aGvHD = acute graft-versus-host-disease; CI = Confidence Interval; HR = Hazard Ratio; * = multivariate analyses adjusted for the following potential cofounders – age, sex, center, underlying diagnosis, conditioning regimen, donor sex, stem cell source, and donor type		

Supplementary Figure 1.

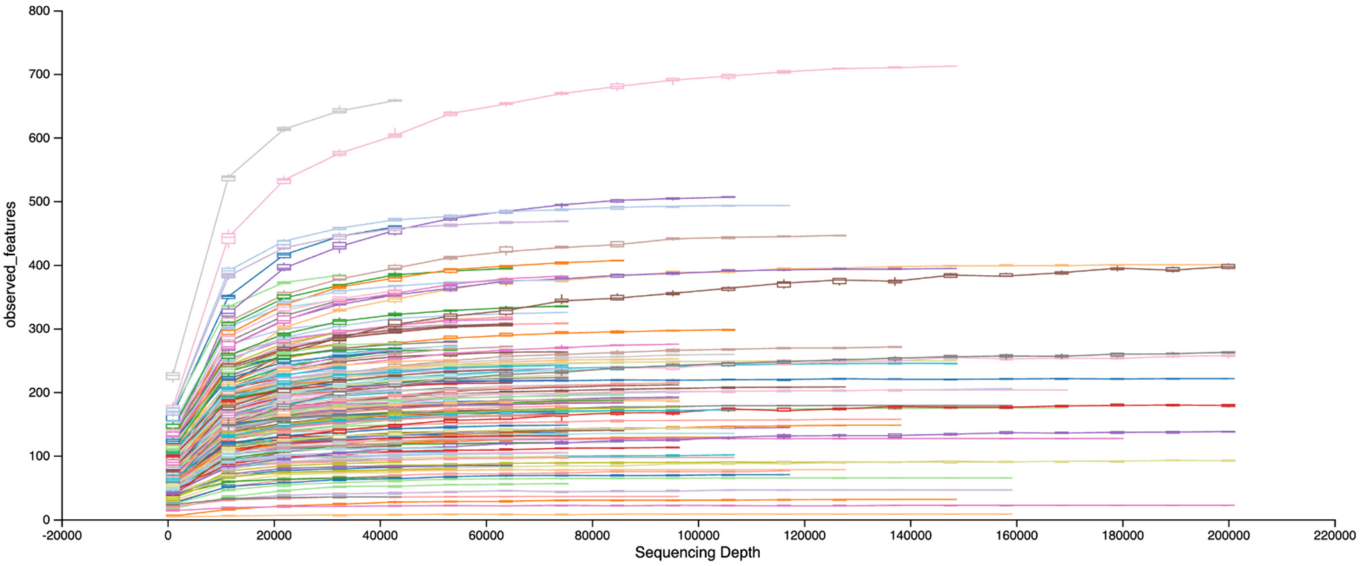

Supplementary Figure 1. Rarefaction curves showing the number of observed features (ASVs) as a function of sequencing depth for all stool samples included in the study. Each line represents an individual sample. ASVs = amplicon sequence variants.

Supplementary Figure 2

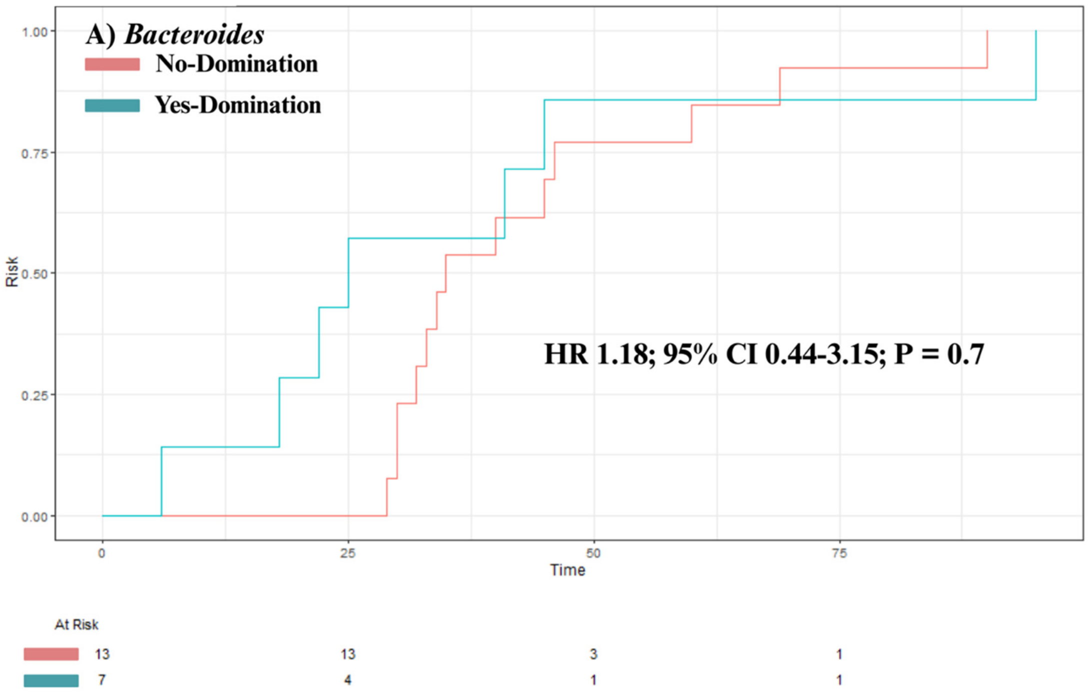
A) Bacteroides
No-Domination
Yes-Domination
HR 1.18; 95% CI 0.44-3.15; P = 0.7
Risk
1.00
0.75
0.50
0.25
0.00
0
25
50
75
Time
At Risk
13 13 3 1
7 4 1 1

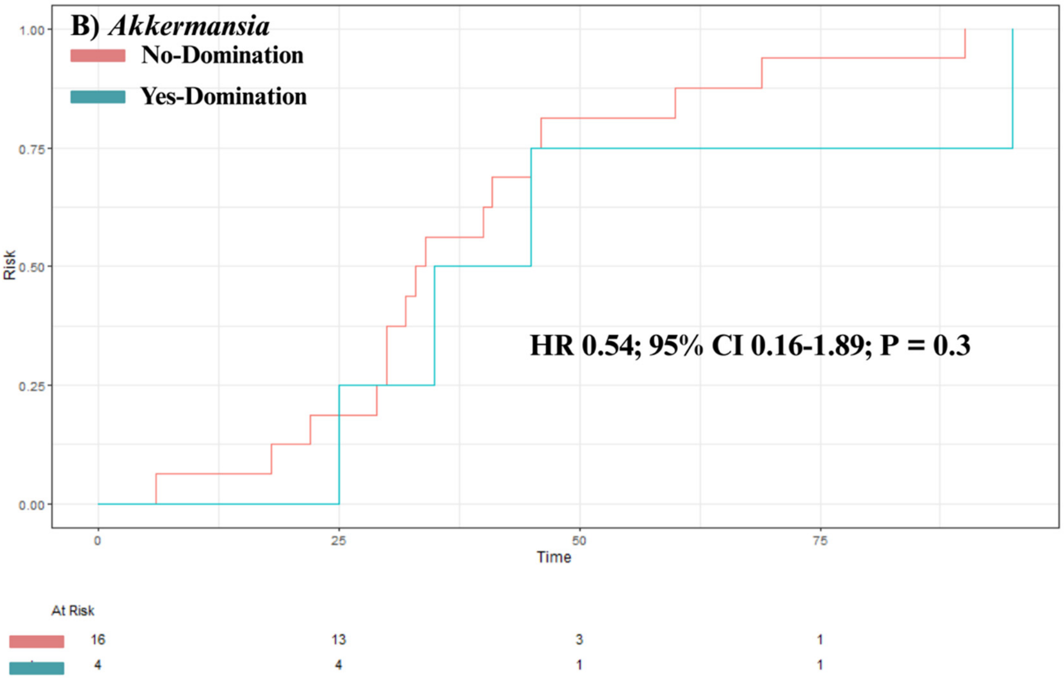
B) Akkermansia
No-Domination
Yes-Domination
HR 0.54; 95% CI 0.16-1.89; P = 0.3
Risk
1.00
0.75
0.50
0.25
0.00
0
25
50
75
Time
At Risk
16 13 3 1
4 4 1 1

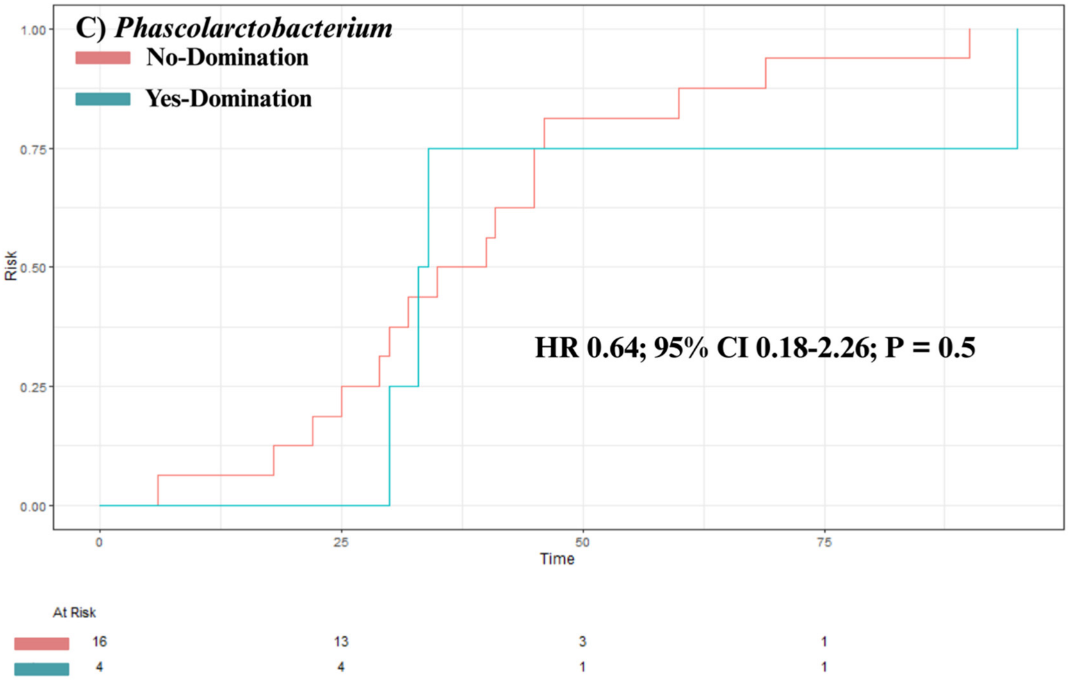

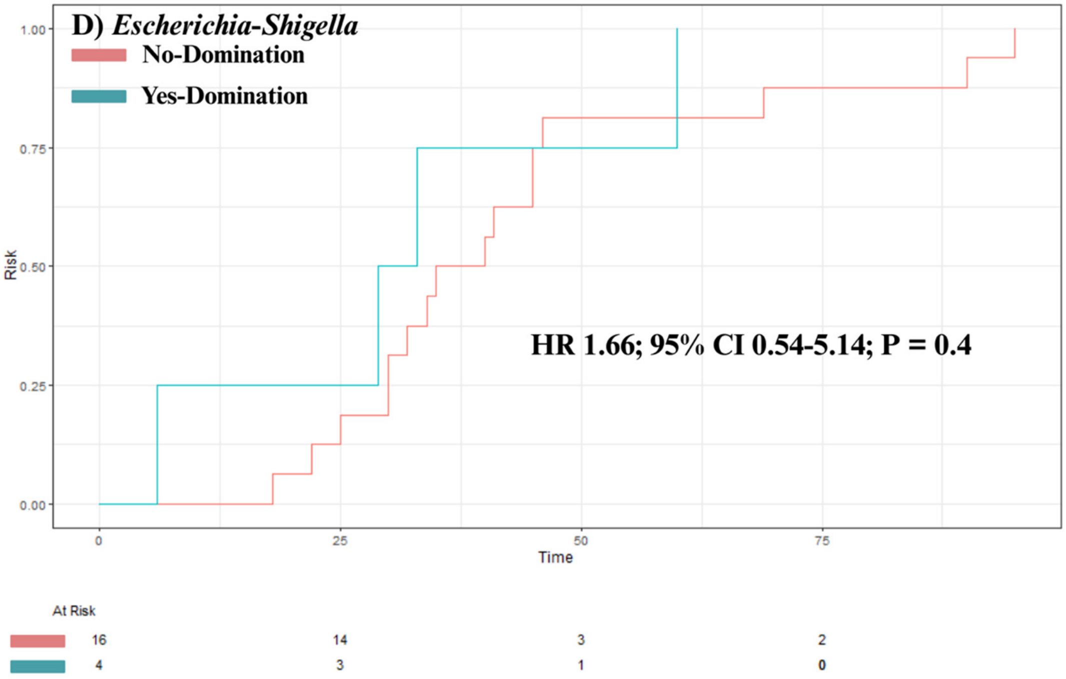

Supplementary Figure 2. The impact of intestinal domination on the cumulative incidence of severe aGvHD. **A)** *Bacteroides*. **B)** *Akkermansia*. **C)** *Phascolarctobacterium*. **D)** *Escherichia-Shigella.* HR = Hazard Ratio. CI = Confidence interval.

Supplementary Figure 3

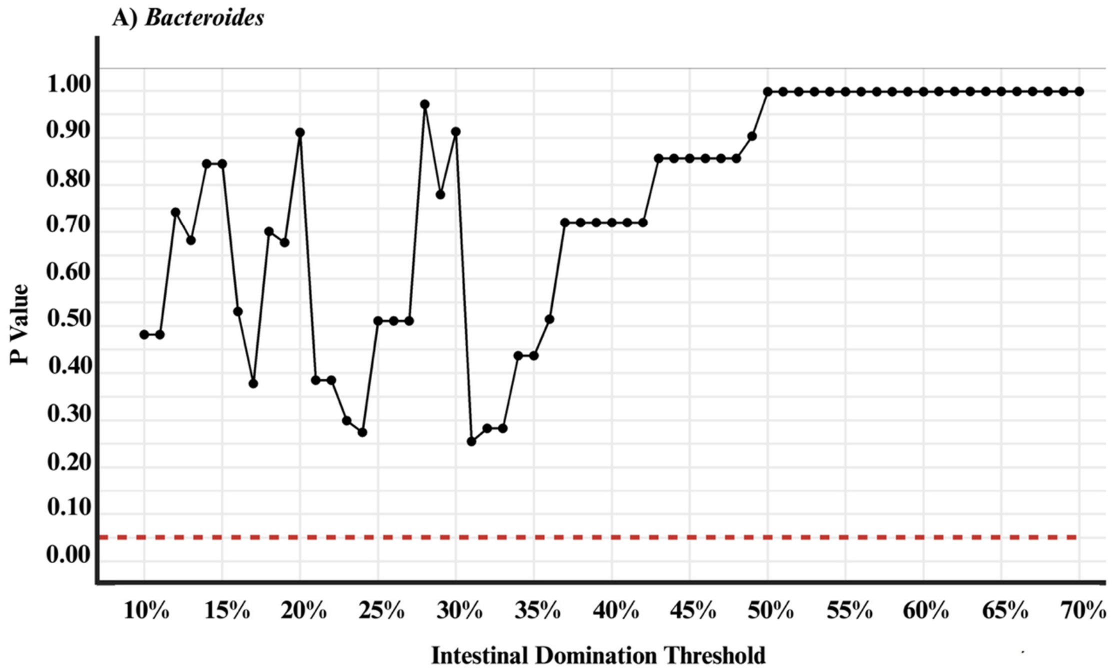

B) *Akkermansia*

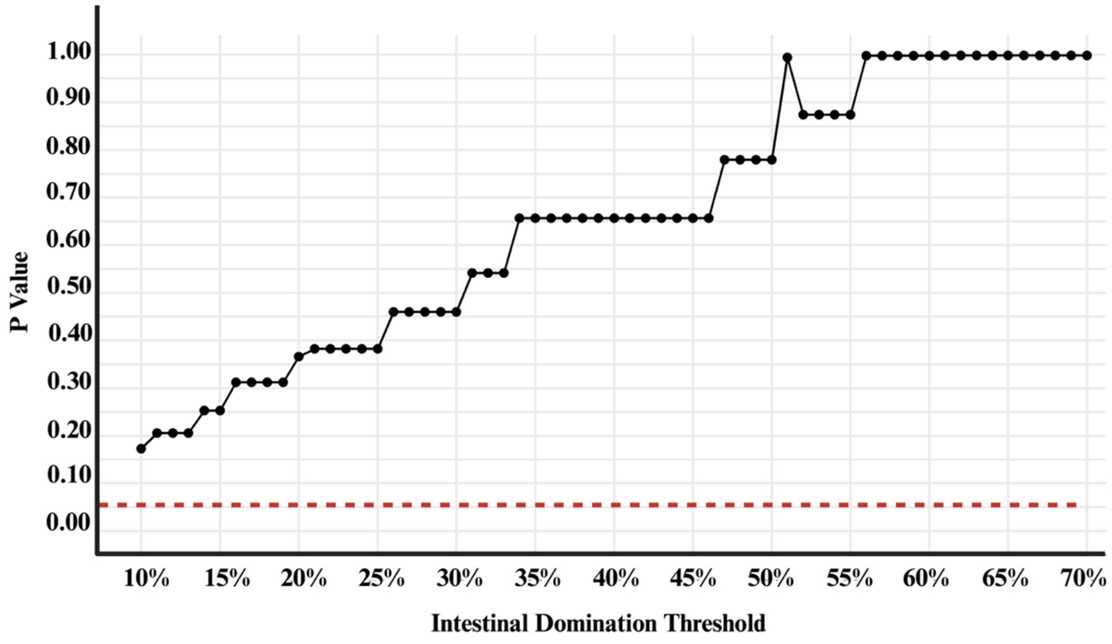

C) *Phascolarctobacterium*

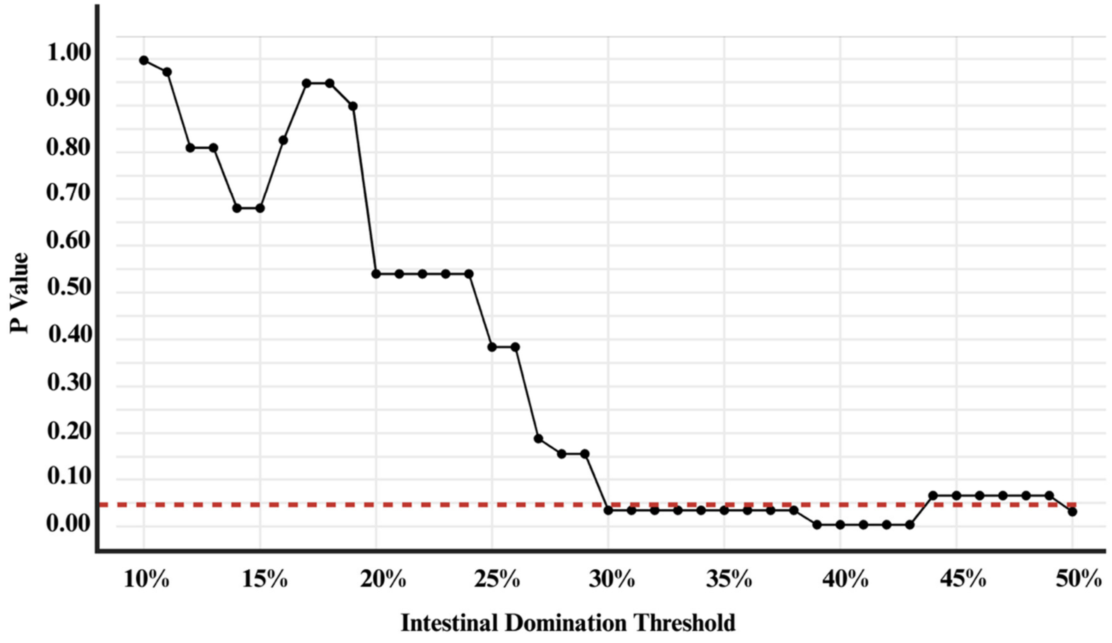

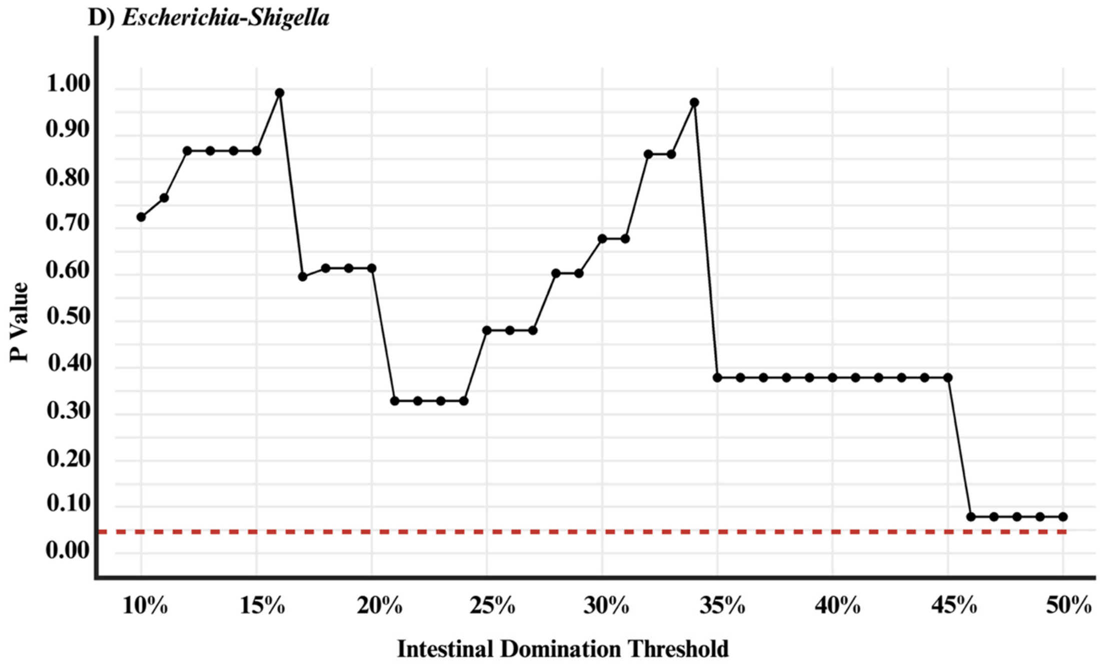

Supplementary Figure 3. Sensitivity analysis to identify the optimal cutoff value for defining intestinal domination in relation to overall survival. (A) *Bacteroides*. (B) *Akkermansia*. (C) *Phascolarctobacterium*. (D) *Escherichia-Shigella*. The y-axis represents the p-value of the Cox regression model for each cutoff tested. The red line represents the significance threshold (P = 0.05).

Supplementary Methods – Predictors of Intestinal Domination

Patients were stratified according to the presence or absence of intestinal domination by any genera or by the four most common genera in any time point. Potential predictors of intestinal domination were selected based on prior studies and included: 1) age, 2) sex, 3) conditioning regimen, 4) stem cell source, 5) TBI (total body irradiation)-containing regimen; and 6) underlying diagnosis (22,23). Similar to prior studies, the underlying diagnosis was classified as acute leukemia vs. others (22,23). The conditioning regimens were classified as 1) myeloablative, 2) nonmyeloablative, and 3) reduced-intensity conditioning per standardized definitions (24). Due to data collection limitations, this study was not able to consider

antibiotics and prior infections as potential predictors of intestinal domination (22,23). However, antibiotic practices in each center are shown in **Supplementary Table 5**.

Supplementary Table 5. Antibiotic Practices in Each Institution

Center	Antibiotic Prophylaxis	First-line Neutropenic Fever	Alternative in Penicillin-allergic Patients
**HB-FUNFARME**	Levofloxacin	Piperacillin-Tazobactam	Meropenem
**HAC**	Levofloxacin (Adults)<br>None (Children)	Piperacillin-Tazobactam	Cefepime
**HCB**	None	Cefepime	Meropenem
**BP**	None	3rd Generation Cephalosporins	Meropenem

BP = Hospital Beneficencia Portuguesa de Sao Paulo, HB-FUNFARME = Hospital de Base of Fundacao Faculdade Regional de Medicina; HAC = Hospital Amaral Carvalho; HCB = Hospital de Cancer de Barretos.
